# Penicillin-binding Proteins (PBP) and Lmo0441 (a PBP-like protein) play a role in Beta-lactam sensitivity of *Listeria monocytogenes*

**DOI:** 10.1186/1757-4749-1-23

**Published:** 2009-12-15

**Authors:** Sébastien Van de Velde, Stéphane Carryn, Françoise Van Bambeke, Colin Hill, Paul M Tulkens, Roy D Sleator

**Affiliations:** 1 Unité de pharmacologie cellulaire et moléculaire, Université catholique de Louvain, Bruxelles, Belgium; 2 Eumedica Pharmaceuticals sa, Manage, Belgium; 3 Alimentary Pharmabiotic Centre, University College Cork, Cork, Ireland; 4 Department of Biological Sciences, Cork Institute of Technology, Rossa Avenue, Bishopstown, Cork, Ireland

## Abstract

While seven penicillin-binding proteins (PBPs) or PBP-like proteins have been identified either by radiolabelled penicillin binding studies or genomic analysis, only PBP3 has been considered of interest for Beta-lactams activity against *Listeria monocytogenes*. Herein we reveal that both PBP4 and Lmo0441 (a PBP-like protein) play a direct role in cephalosporin activity in *L. monocytogenes *while PBP4 additionally has a protective affect against both penicillin and carbapenem.

## Findings

The Gram-positive foodborne pathogen *Listeria monocytogenes *is a causative agent of gastroenteritis [1] and in severe cases, listeriosis, which ranges from a mild flu-like illness to meningitis in non-pregnant individuals, or as infection of the foetus in pregnant women [2]. Ampicillin, either alone or in combination with gentamicin, remains the gold standard treatment for *L. monocytogenes *infection [3]. Conversely, cephalosporins are usually poorly active and thus not recommended in the treatment of listeriosis [4,5]. This dissociation in susceptibilities between β-lactams has been rationalized by the observation that cephalosporins have poor affinity for PBP3 compared to penicillin, suggesting that PBP3 is the primary lethal target for β-lactams in *L. monocytogenes *[6]. However, at least five different PBPs have been identified in *L. monocytogenes *based on their ability to bind radioactive penicillin [6,7]. Moreover, genome analysis revealed seven distinct genes with homologies to PBPs in *L. monocytogenes *[8]. With the exception of *pbpB*, each of the remaining genes has been disrupted by insertional mutagenesis [9] without loss of cell viability, suggesting that they are not critically required for normal synthesis of cell wall peptidoglycan. Based on a previous study by Guinane et al., [9] in which an increase in the activity of cephalosporins was observed for *L. monocytogenes *disrupted in *lmo2229 *and *lmo0441*, we investigated the role of their corresponding proteins (PBP4 and Lmo0441 respectively) in protecting the pathogen against a penicillin (ampicillin, AMP), a carbapenem (meropenem, MEM) and a cephalosporin (cefuroxime, CFX).

**Figure 1:**
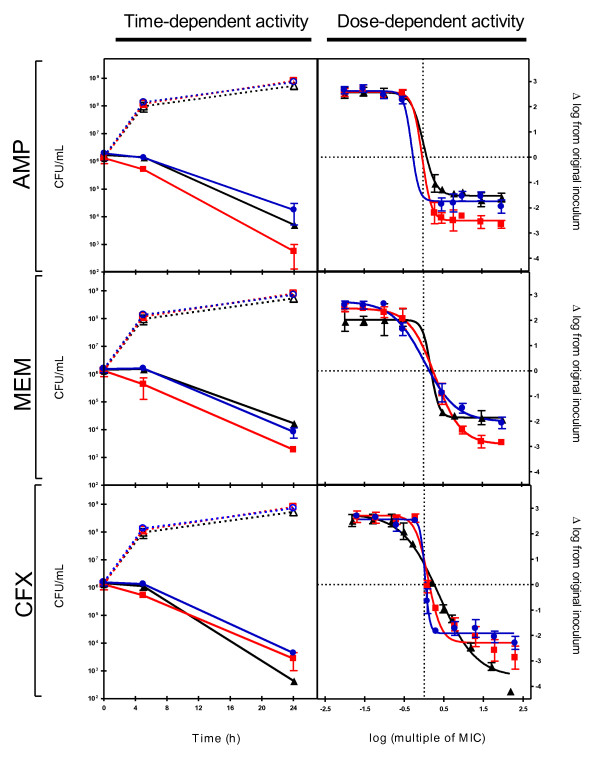
**Activities of ampicillin (AMP), meropenem (MEM) and cefuroxime (CFX,) were tested against EGDe wild-type (triangle), ΔPBP4 (squares) and ΔLmo0441 (circle)**. Left panel shows bacterial growth (open symbols, discontinuous lines) and antibiotic activity (closed symbols and continuous lines) during 24h at 100 times MIC in TSB broth. Right panel shows, at 24h, the dose response curves of the 3 same antibiotics in TSB broth. Curve fitting and statistical analyses were performed using GraphPad Prism for windows version 4.03 and GraphPad Instat version 3.01 (GraphPad® Software, San Diego, CA).

The growth rates for wild-type and mutant strains, together with time profiles for the activity of AMP, MEM and CFX at 100 times their MIC were tested over a 24 h period in TSB broth (Fig. 1, left panel). No significant differences in the growth rates were observed between wild type and both mutant strains. However, this effect is only observed after an incubation of 24 h, the ΔPBP4 strain appeared significantly more sensitive than the wild type to both AMP and MEM, a phenotype not observed for the ΔLmo0441 strain. In contrast, both PBP mutant strains appeared less sensitive to CFX, after 24h, when compared to the wild type strain, an unexpected result given their MICs.

To better understand the role of PBP4 and Lmo0441 proteins, full dose-response experiments were run with AMP, MEM and CFX using a wide range of concentrations and are presented in Fig. 1, right panel. Data collected were used to determine the Hill function regression parameters allowing the determination of the relative drug efficacy (E_max_) presented in Table 1. An increase of drug efficacy was observed for AMP and MEM against the ΔPBP4 strain (1.64 and 1.58 times the E_max _of the parental wild-type strain respectively); an effect which could not have been predicted from MIC measurements; while E_max _of AMP and MEM were not affected in the ΔLmo0441 strain. In contrast to the results obtained for AMP and MEM, both PBPs mutant strains were affected by the activity of CFX and in this case, a decrease of drug efficacy was observed with the use of ΔPBP4 and ΔLmo0441 strains (0.63 and 0.53 times the E_max _of the wild-type strain respectively).

**Table 1:** MIC measurements, regression parameters^a ^and statistical analysis of the dose-response curves illustrated in Figure 1

	AMP			MEM			CFX		
	
	MIC (mg/L)	E_max_^b ^(CI)^c^ Δlog from original inoculum	R^2^	MIC (mg/L)	E_max_^b ^(CI)^c^ Δlog from original inoculum	R^2^	MIC (mg/L)	_max_^b ^(CI)^c^ log from original inoculum	R^2^
**EGDe WT**	0.125	-1.53 (-1.7 to -1.3)	0.97	0.0625	-1.85 (-2.2 to -1.5)	0.95	8	-3.65 (-4.2 to -3.1)	0.97
**ΔPBP4**	0.125	-2.51 (-2.7 to -2.3)^d^	0.98	0.0625	-2.92 (-3.3 to -2.5)^ d^	0.98	2	-2.29 (-2.6 to -1.9)^ d^	0.93
**ΔLmo0441**	0.125	-1.74 (-1.9 to -1.5)	0.97	0.0625	-2.02 (-2.5 to -1.6)	0.97	2	-1.92 (-2.2 to -1.7)^ d^	0.95

Ampicillin (AMP), meropenem (MEM) and cefuroxime (CFX) MICs measurements for the parental strain EGDe and ΔPBP4 and ΔLmo0441 mutant strains. MICs of AMP and MEM, with a respective value of 0.125 and 0.0625 mg/L, did not differ between wild type and mutant strains. However, there is a 4-fold increase in MIC of CFX for both mutant strains, which passes from 8 mg/L to 2 mg/L, a finding which is in agreement with previous observations [9].

^a ^Regression parameters are based on the Hill equation (using a variable slope)

^b ^Concentration causing maximal activities (E_max _values), as obtained from the Hill equation. Values are expressed as a multiple of the respective MIC for a given strain.

^c ^CI - Confidence Intervals (95%)

^d ^Significantly different (*p *< 0.05) from the wild type strain as calculated by F-test

**Figure 2:**
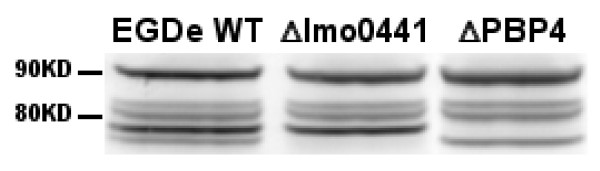
**Western Blot analysis of PBPs was performed as described previously **[10]**with minor modifications**. 10 ml of log phase bacteria (OD_660 _nm =0.5) were exposed to Bocillin FL (25 μg) for 30 min at 37°C. Bacteria were then harvested by centrifugation, washed four times with phosphate-buffered saline, and lysed by freeze-thawing process. Proteins were separated by 1D SDS-PAGE, and the acyl-enzyme complex was detected (excitation: 488nm, emission: 520nm) with a fluorescent scanner (Typhoon 9410, GE Healthcare). Mutagenesis results in a loss of bands of approximately 75 and 78 KDa which correspond to the molecular weight of PBP4 and Lmo0441 proteins respectively.

In conclusion then, the construction of PBP mutant strains ΔPBP4 and ΔLmo441 (Fig. 2) displayed no obvious growth defects in *L. monocytogenes*, suggesting that the individual PBPs are most likely not essential for growth under normal growth conditions. Furthermore, we demonstrate a role for PBP4 in penicillin and carbapenem activities in *L. monocytogenes*. Indeed, dose-response experiments over a wide concentration range reveals an increase in sensitivity of the ΔPBP4 strain for both AMP and MEM compared to the parental wild-type strain. This observation, which could not have been inferred from MIC measurements alone, underscores the importance of accurate techniques to predict antibiotic activity. Moreover, this effect appears specific to the ΔPBP4 strain and is not apparent in the ΔLmo0441 mutant. The observed protective effect of PBP4 against penicillin and carbapenem activities may result from decreased competition between PBPs or from the loss of cooperative action with other PBPs for the formation of the cell wall peptidoglycan.

This study also reveals that loss of PBP4 or Lmo0441 proteins allows a decrease of maximal activity of cephalosporin, a class of antibiotics which, due to their poor affinity for the PBP3, are not particularly effective against *L. monocytogenes *[6]. This observation, which runs contrary to what was observed for AMP and MEM may be explained by the fact that PBP3 may increase its relative action in peptydoglycan synthesis when PBP4 or lmo0441 are deleted: conferring resistance to cephalosporin. Thus, even if PBP3 remains the principal target of β-lactam antibiotics, as is the current dictum, PBP4 and Lmo0441 also appear to play an important role in the activity of β-lactam antibiotics.

## Competing interests

The authors declare that they have no competing interests.

## Authors' contributions

SVDV, PMT, CH and RDS conceived of the study and drafted the manuscript. SVDV, SC and FVB carried out the work. All authors read the manuscript and approved the final draft.
